# Fifth Report of the New York Ophthalmic and Aural Intitute

**Published:** 1875-05

**Authors:** 


*Fifth Report of the New York Ophthalmic and Aural Institute*, from May 1st, 1873, to Dec. 31, 1874.

Total number of Eye and Ear operations performed—725.

